# Association of FMO3 Variants and Trimethylamine N-Oxide Concentration, Disease Progression, and Mortality in CKD Patients

**DOI:** 10.1371/journal.pone.0161074

**Published:** 2016-08-11

**Authors:** Cassianne Robinson-Cohen, Richard Newitt, Danny D. Shen, Allan E. Rettie, Bryan R. Kestenbaum, Jonathan Himmelfarb, Catherine K. Yeung

**Affiliations:** 1 Kidney Research Institute, Division of Nephrology, Department of Medicine, University of Washington, Seattle, Washington, 98195, United States of America; 2 Department of Pharmacy, School of Pharmacy, University of Washington, Seattle, Washington, 98195, United States of America; 3 Department of Medicinal Chemistry, School of Pharmacy, University of Washington, Seattle, Washington, 98195, United States of America; The University of Tokyo, JAPAN

## Abstract

Elevated levels of circulating pro-atherogenic uremic solutes, particularly trimethylamine N-oxide (TMAO), have been implicated in cardiovascular disease development in patients with chronic kidney disease (CKD). TMAO is generated from trimethylamine (TMA) via metabolism by hepatic flavin-containing monooxygenase isoform 3 (FMO3). We determined the functional effects of three common FMO3 variants at amino acids 158, 308, and 257 on TMAO concentrations in a prospective cohort study and evaluated associations of polymorphisms with CKD progression and mortality. Each additional minor allele at amino acid 158 was associated with a 0.38 μg/mL higher circulating TMAO (p = 0.01) and with faster rates of annualized relative eGFR decline. Participants with 0, 1 and 2 variant alleles averaged an eGFR loss of 8%, 12%, and 14% per year, respectively (p-for trend = 0.05). Compared to participants with the homozygous reference allele, heterozygous and homozygous variant participants had a 2.0-fold (95% CI: 0.85, 4.6) and 2.2-fold (95% CI: 0.89, 5.48) higher risk of mortality, respectively (p-for-trend = 0.04). No associations with clinical outcomes were observed for allelic variants at amino acids 257 or 308. Understanding the contribution of genetic variation of FMO3 to disease progression and all-cause mortality can guide recommendations for diet modification or pharmacotherapy in CKD patients at increased risk of adverse outcomes.

## Introduction

Chronic kidney disease (CKD) is associated with increased risks of cardiovascular events and a shortened lifespan, independent of diabetes mellitus, hypertension, and other traditional risk factors [1–3]. The metabolite trimethylamine N-oxide (TMAO), as well as TMAO precursors, choline, and betaine accumulate in CKD and are considered putative uremic toxins, based on associations with coronary heart disease and mortality [4, 5]. Moreover, metabolomic studies have identified TMAO as a cardiovascular risk factor among patients undergoing elective cardiac evaluation [6].

Trimethylamine (TMA), TMAO, and choline are generated from dietary phosphatidylcholine, which is abundant in high-fat foods. Intestinal flora degrade phosphatidylcholine to choline, most of which is subsequently oxidized to betaine aldehyde [7]. The remainder of dietary choline is converted to TMA, which is rapidly metabolized primarily by hepatic flavin-containing monooxygenase isoform 3 (FMO3) to generate the oxidized product TMAO [8]. TMAO is then excreted efficiently by healthy kidneys [9], with normal subjects excreting more than 18 μmol TMA/μmol creatinine daily with a urinary TMA:TMAO ratio of less than 1:20 [10, 11].

Genetic variation in the FMO3 enzyme modifies the production of TMAO, which may be particularly consequential for CKD patients. Differences in TMAO concentrations or clinical outcomes may not manifest in patients without moderate or severe kidney impairment due to rapid elimination of metabolites. Rare FMO3 gene variants markedly diminish catalytic activity and result in a phenotype known as symptomatic trimethylaminuria, or “fish odor syndrome”, which is anecdotally associated with hypertension and affective disorders [12]. Non-dominant variants of FMO3 are common, with as many as 50% of individuals in certain populations possessing a non-synonymous FMO3 allelic variant [13, 14]. The effect of the most common variant, FMO3 p.158 E>K is controversial, with reports of mildly decreased [15–17] or no effect on activity in healthy subjects. We have reported that the recombinantly expressed FMO3 158K variant exhibits a 35% increase in catalytic efficiency (kcat/Km) compared with the wild-type 158E variant for both amine and sulfide containing substrates [18]. Some of this inconsistency may arise from modest linkage with FMO3 p.308 E>G, an allelic variant that confers reduced enzyme activity both in vitro and in vivo. The less common FMO3 p.257V>M variant has also been shown to have slightly reduced activity in vivo [13]. Differences in FMO3 activity and subsequent TMAO production in healthy subjects may be masked by rapid and efficient renal excretion; changes in FMO3 activity may be revealed in the setting of CKD due to impaired TMAO excretion. Understanding the contribution of genetic variation of FMO3 to disease progression and all-cause mortality can guide recommendations for diet modification or pharmacotherapy in CKD patients at increased risk of adverse outcomes.

## Methods

Chemicals and Reagents: Trimethylamine N-oxide dehydrate was obtained from SigmaAldrich (St. Louis, MO, USA). Choline chloride and betaine monohydrate were obtained from Acros Organics (NJ, USA). Deuterated internal standards (d9-TMAO and d9-choline) were obtained from Cambridge Isotope Laboratories (Cambridge, MA, USA). Optima LC-MS grade water, acetonitrile (ACN) and methanol (MeOH) were purchased from Fisher Scientific (Pittsburgh, PA, USA). A pooled plasma sample from healthy male subjects (HS-422) was obtained from SigmaAldrich (St. Louis, MO, USA). DC Mass Spect Gold (Cat #MSG- 4000), an ultra-low analyte human serum, was obtained from Golden West Biologicals (Temecula, CA, USA)

Study population: The Seattle Kidney Study (SKS) is an ongoing clinic-based cohort study of individuals with CKD based in Seattle, WA [19–21]. The SKS began recruiting participants in 2004 from outpatient nephrology clinics at Harborview Medical Center and the Veterans’ Affairs Puget Sound Health Care Center. Eligibility criteria are age > 18 years, eGFR ≤ 90 ml/min/1.73m^2^ or a urinary protein to creatinine ratio of > 30 mg/g. Exclusions for entry into the cohort include current dialysis, kidney transplantation or expectation of starting renal replacement therapy, or of leaving the area within 3 months. Pre-menopausal women were not excluded from participation in the study. The University of Washington institutional review board approved the study, and all participants provided informed consent.

Genotyping: Genotyping was performed on DNA extracted from whole blood samples that were obtained from study subjects at baseline study enrollment. Genotyping was performed by the Functional Genomics Laboratory of the NIEHS Center for Ecogenetics and Environmental Health at the University of Washington, using commercially available TaqMan Detection System-based genotyping assays (Applied Biosystems, Inc., Hercules, CA). Subjects were evaluated for *FMO3* g.15167G>A (FMO3 p.158 E>K), *FMO3* g.18281G>A (FMO3 p.257V>M), and *FMO3* g.21446A>G (FMO3 p.308E>G): *rs2266782*, *rs1736557*, and *rs2266780*, respectively. Details of genotyping methods, including quality control procedures, have been previously described [22, 23].

Sample processing and analysis: Human plasma samples (30 μl) were subjected to protein precipitation with acetonitrile (ACN) at an ACN: plasma ratio of 6:1 (v/v). The supernatant was further diluted with 86% ACN (final plasma dilution 1/20), from which an aliquot (20 μl) was injected onto the LC column with a CTC LEAP Technologies HTS PAL autosampler at 100°C. Anonymized patient samples were run as duplicates.

The analytical measurement of the products of phosphatidylcholine metabolism: choline and TMAO in human plasma was performed by stable isotope assay dilution liquid chromatography-mass spectrometry (LC-MS). Separation of the analytes and their respective deuterated internal standards in a single run was accomplished by hydrophilic interaction chromatography (HILIC) over an underivatized silica column (WATERS Atlantis: 2.1 x 100 mm, 3 μm) using a Finnigan Surveyor HPLC pump with a flow rate set at 400 μl/min. Analytes were eluted by increasing the aqueous composition of mobile phase under constant salt conditions. The LC mobile phases were: (A) 100 mM NH_4_HCOO, 0.5% HCOOH pH 3.4 (B) ACN and (C) H_2_0. Initial conditions were 5% A, 87.5% B, and 7.5% C. The percentage of (A) remained constant over the entire run. After 2 minutes, the percentage of (C) was raised stepwise to 15% and then in a gradient fashion from 15% to 35% from 2.01–7.5 min. The column was washed for 2 minutes at 35% C, then re-equilibrated to initial conditions for 4.5 minutes. During column re-equilibration, a second LC pump delivered a H_2_0:ACN (95:5 v/v) wash just over the ionization needle at the mass spectrometer to flush salt.

A Thermo Finnigan TSQ Quantum Ultra triple quadrupole mass spectrometer was used to detect analytes in positive ion mode with selected reaction monitoring. The following selected ion transitions were monitored: 104.1 to 60.2 m/z and 104.1 to 58.2 (choline); 113.2 to 69.2 m/z (d-choline); 76.0 to 58.1 m/z (TMAO); 85.2 to 66.2 m/z and 85.2 to 68.2 m/z (d-TMAO). Vaporizer temperature was set at 400°C. Collision energies for choline and TMAO were 16 and 32, and 15 (arbitrary units for TSQ Quantum Ultra), respectively. Collision (Q2) gas pressure was 1.5 mTorr. Signal output from the mass spectrometer was captured and processed with ThermoFinnigan XCaliber software version 2.2.

Calibration curves were generated in MSG 4000 (low analyte human plasma), using known final concentrations of analytes (0, 3.75, 7.5, 15, 30, 60, 120, 240, 480 ng/ml) and a fixed concentration (120 ng/ml) of each corresponding internal standard. Deuterated internal standards (d9-TMAO, and d9-choline) were added to all samples before processing. Working stocks (1 mg/ml) of internal standards and analytes were made in ACN:MeOH (75:25 v/v) and kept at -70°C. Chromatographic peak areas for a given analyte and its corresponding internal standard were integrated and the data expressed as a peak area ratio of analyte/ internal standard vs nominal dose from which quantification of the amount of analyte in plasma samples was extrapolated. Retention times for choline and TMAO were 5.4, and 6.8 min, respectively. Peaks were well separated and there was no interference from other compounds. Recovery of analyte following protein precipitation was greater than 95%.

Outcome ascertainment: Serum creatinine and cystatin C measurements were performed annually in SKS as previously described [24]. We calculated estimated GFR annually using the combined cystatin and creatinine-based CKD-EPI equation [25].

The primary renal outcome was the relative annualized change in kidney function, defined by the exponentiated slope of log-transformed eGFR values [24].

In order to ascertain all-cause mortality, study coordinators identified death events from proxies during surveillance calls, during scheduling calls for annual visits, by screening medical records, and by linkage with the social security death index.

Measurement of Covariates: Serum, plasma, and urine were stored at -70°C until analysis. Concentrations of low-density lipoprotein (LDL) cholesterol, high-density lipoprotein (HDL) cholesterol and C-reactive protein (CRP) were measured in serum. Three seated blood pressure measurements were recorded using an automated sphygmomanometer; the average of the last two readings was retained for analysis. Smoking status was ascertained via baseline lifestyle questionnaires.

Statistical Analyses: Baseline descriptive statistics on demographics, medical history and clinical, laboratory and lifestyle characteristics were examined according to FMO3 p.158 E>K variant and overall, and are presented as mean and standard deviation (SD) for continuous variables and number are proportion for categorical variables.

Mean TMAO concentrations are presented by genotype as median (IQR). The association of genotype with TMAO concentration was evaluated using linear regression, with TMAO concentration as the dependent variable, and genotype as predictors, under an additive genetic model. Beta coefficients from these models can be interpreted as the difference in TMAO concentration associated with each additional copy of the minor allele, holding other model covariates constant. Covariates in the first adjustment model were age, sex, race and estimated eGFR. A second model additionally adjusted for circulating choline concentrations.

Generalized estimating equations (GEE), accounting for within-participant clustering across time, were used to determine if the annualized relative or absolute change in eGFR differed across FMO3 variant or TMAO tertiles, after adjusting for potential confounding variables [26]. For the association of genotype with change in eGFR, we adjusted for age, race (white, black, other) and sex. For the association of TMAO tertile with change in eGFR, we adjusted for age, race and sex in a first model and additionally adjusted for systolic blood pressure, low and high-density lipoprotein cholesterol concentrations, smoking status (never, former, current), and log-transformed CRP in a second model. For the mortality endpoint, participants were considered at risk from the date of their baseline exam until the date of death from any cause, loss to follow-up or end of data collection, defined as January 1^st^, 2012. We calculated unadjusted incidence rates as the number of events divided by person-years at risk and used Cox proportional hazards regression to estimate the relative hazard of death after adjustment for potential confounding factors. For the association of genotype with mortality, we adjusted for participant age, race, gender, and eGFR. For the association of TMAO tertile with mortality, we adjusted for age, race and sex in a first model and additionally adjusted for systolic blood pressure, low and high-density lipoprotein cholesterol concentrations, smoking status (never, former, current), log-transformed C-reactive protein, and baseline eGFR in a second model.

Analyses were performed using STATA release 13.1 (StataCorp, College Station, TX).

## Results

**Fig 1:**
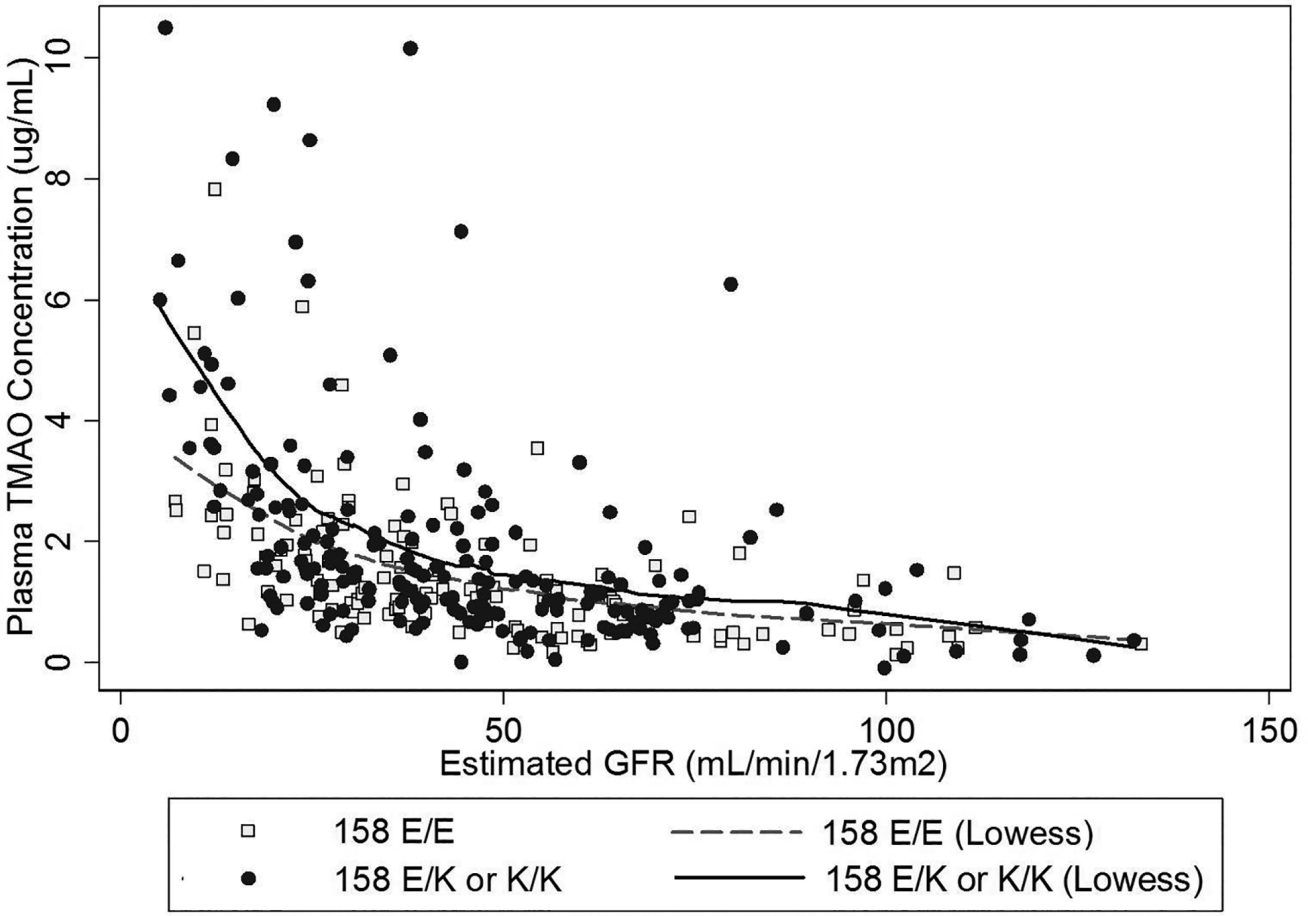
Scatterplot of plasma TMAO concentration versus estimated GFR for subjects with FMO3 E/E (open square) and FMO3 E/K or K/K (closed circle) allelic variants. TMAO concentration increases as eGFR decreases, with greater rate of increase observed with eGFR < 40 mL/min/1.73m^2^. Dashed line indicates the Lowess curve for reference allele homozygous subjects (158 E/E). Dotted line indicates the Lowess curve for heterozygous or variant homozygous subjects (158 E/K or K/K).

Baseline Characteristics and plasma TMAO concentrations: The prevalences of the E/E, E/K, and K/K variants at amino acid position 158 in this CKD study population were 39%, 47%, and 14%, respectively (Table 1). For variants at position 257 and 308, the prevalences of the minor allele were 5.8% and 15.5%, respectively. The mean (standard deviation) plasma TMAO concentration among the full cohort was 1.76 (1.94) μg/mL. Plasma concentrations of TMAO were progressively higher with lower estimated GFRs, particularly for eGFRs less than 40 mL/min/1.73m^2^ (Fig 1).

**Table 1:** Demographics of study subjects according to FMO3 p.158E>K genetic variant.

Mean ± SD or n (%)	All subjects (n = 339)	E/E (n = 132)	E/K (n = 161)	K/K (n = 46)
Age at enrollment (years)	57.3 ± 13.5	55.7 ± 12.9	58.7 ± 13.9	57.9 ± 13.4
BMI (kg/m^2^)	31.7 ± 7.8	32.5 ± 7.8	31.2 ± 8.1	30.7 ± 7.7
eGFR CKD-EPI (mL/min/1.73m^2^)	44.2 ± 23	47.4 ± 28.3	46.8 ± 26.2	42.7 ± 22.3
Gender (female)	107 (31)	43 (32.6)	46 (28.6)	14 (30.4)
Race				
American Indian/Native Alaskan	7 (2.0)	2 (1.5)	5 (3.1)	0 (0.0)
Asian/Pacific Islander	10 (2.9)	6 (4.6)	1 (0.6)	3 (6.5)
Black	89 (25.5)	38 (28.8)	36 (22.4)	15 (32.6)
White	225 (64.5)	80 (60.6)	111 (68.9)	26 (56.5)
Other	18 (5.2)	6 (4.6)	8 (5.0)	2 (4.4)
Prevalent Diabetes	170 (48.7)	70 (53.0)	75 (46.6)	22 (47.8)
Prevalent Hypertension	328 (93.9)	127 (96.2)	149 (92.6)	43 (93.5)

FMO3 genotype and circulating TMAO concentrations in CKD: A greater number of minor alleles at amino acids 158 and 308 were associated with higher plasma TMAO concentrations (Table 2). At the 158 locus, each additional variant allele was associated with an estimated 0.38 μg/mL greater circulating TMAO concentration (p = 0.01) after adjustment for age, race, gender, and eGFR. Plasma TMAO concentrations were consistently higher among participants who had the E/K or K/K variants at the 158 locus compared to those who had the E/E variant across the distribution of eGFR, with divergence between individuals homozygous for the reference allele and those heterozygous or homozygous for the variant allele below eGFR = 40 mL/min/1.72m^2^ (Fig 1). At the 308 locus each additional minor allele was associated with an estimated 0.46 μg/mL greater circulating TMAO concentration (p = 0.03) after adjustment. In contrast, additional minor alleles at the 257 locus tended towards lower TMAO concentrations; however, these differences were not statistically significant. Associations of genetic variants with plasma TMAO concentrations were unaltered by further adjustment for concentrations of substrate choline.

**Table 2:** FMO3 allelic variants vs plasma TMAO concentration.

		TMAO concentration (μg/mL)	
FMO3 variant	n	Median	IQR (25%, 75%)
158			
E/E	131	1.14	0.59, 1.91
E/K	161	1.34	0.72, 2.22
K/K	46	1.29	0.73, 2.39
Adjusted* β (SE) per minor allele, p for trend		0.38 (0.15); p = 0.01	
Adjusted** β (SE) per minor allele, p for trend		0.38 (0.15); p = 0.01	
257			
V/V	300	1.27	0.70, 2.14
V/M	35	1.05	0.80, 1.89
M/M	2	0.97 (mean)	0.18 (SD)
Adjusted* β (SE) per minor allele, p for trend		-0.50 (0.31); p = 0.10	
Adjusted** β (SE) per minor allele, p for trend trend		-0.49 (0.31); p = 0.11	
308			
E/E	240	1.19	0.74, 2.01
E/G	90	1.36	0.73, 2.14
G/G	8	2.05	0.81, 3.16
Adjusted* β (SE) per minor allele, p for trend		0.46 (0.22); p = 0.03	
Adjusted** β (SE) per minor allele, p for trend trend		0.46 (0.22); p = 0.04	

*Adjusted for age, race, sex, eGFR

**Additionally adjusted for choline

FMO3 genotype, kidney disease progression, and mortality: During a median follow-up of 3.3 years (interquartile range, 1.9–4.6 years) the average relative decline in eGFR was 8.5% per year (standard error = 0.015) and there were a total of 45 deaths. Participants who had 0, 1 and 2 variant alleles at the 158 locus averaged an eGFR loss of 8%, 12%, and 14% per year, respectively (Table 3; p-for trend = 0.05). Compared to participants who were homozygous for the reference allele (E/E), heterozygous participants (E/K) and homozygous variant participants (K/K) had a 1.97-fold (95% CI: 0.85, 4.60) and 2.22-fold (95% CI: 0.89, 5.48) greater risk of mortality (p-for-trend 0.04). In contrast, neither the 257 nor 308 genotypes were associated with kidney disease progression or mortality. To investigate whether associations of variation at amino acid 158 with study outcomes were mediated via effects on circulating TMAO and choline concentrations, we conducted sensitivity analyses that added adjustment for these metabolites.

**Table 3:** Association of Genotype and Kidney Disease Progression and all-cause mortality.

FMO3 Variant	Adjusted^†^ mean % change in eGFR per year (95% CI)	Adjusted^†^ mean absolute decline per year, in mL/min/1.73m^2^/yr (95% CI)	Number of deaths	Incidence Rate, per 1000 p-y	Adjusted* HR (95% CI)
158					
E/E	-8.40 (-10.8, -6.00)	-1.70 (-2.62, -0.79)	10	26.1	1.0 (ref.)
E/K	-11.8 (-24.3, +0.75)	-1.57 (-8.14, 5.83)	26	54.4	1.97 (0.85, 4.60)
K/K	-14.1 (-32.2, +3.96)	-4.75 (-11.74, 2.24)	8	62.2	2.22 (0.89, 5.48)
p-for-trend	0.05	0.05			0.040
257					
V/V	-7.07 (-8.80, -2.33)	-1.35 (-2.00, 0.70)	43	49.3	-
V/M	-9.70 (-28.5, +9.11)	-1.15 (-6.34, 3.21)	1	9.34	-
M/M	-12.45 (-79.8, +54.9)	-6.91 (-33.72, 19.89)	0	-	-
p-for-trend	0.07	0.07			
308					
E/E	-6.68 (-8.66, -4.70)	-1.46 (-2.22, -0.74)	26	37.5	1.0 (ref.)
E/G	-13.65 (-26.7, -0.06)	-3.09 (-8.15, 1.96)	16	60.3	1.56 (0.76, 3.23)
G/G	-27.93 (-58.54, +0.03)	-11.91 (-26.63, 2.81)	2	73.6	1.87 (0.59, 5.95)
p-for-trend	0.96	0.963			0.143

^**†**^ Adjusted for age, race and sex.

*Adjusted for age, race, sex and eGFR

Treating the FMO3 158 genotype as a continuous variable (for the p-for-trend estimation) involves coding the genotype as 0, 1, 2 minor alleles (K alleles). With basic adjustment for age, race, and gender, each additional minor allele at 158 was associated with a 2.3% lower difference in annualized relative GFR change (95% CI 0% lower,4.7% lower) and a 1.94 hazard ratio of mortality (95% CI: 0.88, 4.27). After additional adjustment for plasma TMAO and choline concentrations each minor allele was associated with a 2.1% mean difference in annualized GFR change (95% CI 4.5% lower, 0.5% higher, p = 0.085) and a 1.48 hazard ratio of death (95% CI 0.98, 2.22, p = 0.062). The test for trend is the more powerful test for a linear change in effect estimates, and is commonly used as a summary measure of statistical significance for ordered categorical variables. The association of each category of the exposure with the outcome does not have to be significant in order for the trend to be statistically significant.

Plasma TMAO concentrations were not significantly associated with kidney disease progression or all-cause mortality, after adjustment for potential confounders (S1 Table).

## Discussion

In this clinic-based cohort study of participants with moderate-to-severe CKD, we demonstrate for the first time, that FMO3 genotype at amino acid 158, but not 257 or 308, is associated with plasma TMAO concentrations, kidney function decline, and all cause-mortality. However, plasma TMAO itself is not significantly associated with clinical outcomes after adjustment for potential confounders.

Because genetic information is allocated randomly, genetic variation can be used as an effective tool to distinguish potentially causal from non-causal biomarkers through an approach known as Mendelian randomization. Unlike most instances in observational study settings, the described association between FMO genotype and events should not be subject to reverse causality bias, regression dilution bias, or to confounding by other risk factors related to TMAO. Our findings, in conjunction with known associations of TMAO and clinical outcomes, suggest a connection between TMAO in CKD progression and related complications, however, the TMAO concentration may instead be a surrogate measure of variability in FMO3 activity.

The pathophysiologic mechanisms by which TMAO might contribute to cardiovascular disease and decreased GFR remain unclear. To date, TMAO has been considered to be a beneficial and protective osmolyte, that acts as a chemical chaperone to facilitate intracellular protein folding and buffer mutational variation [27]. An indirect pathophysiologic effect of TMAO is evidenced by studies in mice overexpressing FMO3 that showed increases in lipogenesis and gluconeogenesis due to perturbation of PPARα and Kruppel-like factor 15 pathways, with a corresponding protective effect in FMO3 knockout mice [28]. Global microarray studies suggested that these effects were mediated by an FMO3-dependent modulation of adiposity rather than a direct effect of TMAO. Indeed, TMAO may simply be a marker for FMO3 function, and not the causative agent for the observed clinical effects as FMO3 has been reported to be a central regulator of cholesterol balance [29] and may also play a role in regulation of blood pressure. This is supported by a large study (n = 2995) that recently showed an association between the FMO3 p.158E>K variant and essential hypertension, but did not control for renal function or measure plasma concentrations of TMAO in the cohort [30].

The FMO3 gene likely plays a role in normal physiology, and has been under positive evolutionary selection since human populations migrated out of Africa [31]. The substrate specificity of FMO3 is broad, known substrates include very small (e.g. TMA, MW = 59 Da) and moderately sized substrates (e.g. ketoconazole, MW = 531 Da) containing nucleophilic nitrogen, sulfur, or phosphorus atoms. While FMO3 is sometimes considered to be a drug metabolizing enzyme, the primary role of FMO3 appears to be the bioconversion of endogenous substrates, namely TMA, but possibly endogenous regulators of cardiovascular function. FMO3 catalyzes the oxidation of catecholamine or catecholamine-releasing vasopressors, including tyramine, phenylethylamine, adrenaline, and noradrenaline [32, 33]. It is also possible that FMO3 is involved in the bioactivation or catabolism of as yet unidentified cell signaling mediators that play a role in cholesterol balance [29], leading to an increased incidence of cardiovascular disease and mortality.

This study is limited primarily by the small sample size and the limited number of mortality events which prevented the separation of adjudicated cardiovascular deaths from all-cause mortality. All-cause mortality was considered a secondary outcome, and is very preliminary given the small number of events; future studies in a larger cohort with adjudicated cardiovascular deaths will be required to validate these findings. We have only evaluated three loci in the FMO3 gene, and not comprehensively evaluated variation in FMO3 or the effects of multiple simultaneous variants, limiting the interpretation of the study. The results of this study may not be generalizable to healthy subjects who are able to efficiently excrete TMAO, and to subjects receiving hemodialysis. No association between TMAO concentration and all-cause mortality, cardiovascular death or hospitalization was observed in 235 patients receiving hemodialysis [34]; in the HD population, the number of events due to TMAO is likely overwhelmed by the high background levels of mortality and hospitalization. This study is also limited by the lack of assessment of the content and timing of dietary intake by the participants. As TMA, the TMAO precursor, is derived largely from sources of animal protein (meat, fish, eggs), intake of these food products prior to sampling could lead to transient increases in TMA and TMAO.

Finally, understanding the contribution of genetic variation of FMO3 to the risk of disease progression and all-cause mortality in CKD patients can guide personalized recommendations for diet modification (eg., reduction of phosphatidylcholine) or pharmacologic intervention to reduce of TMAO production in patients at increased risk of adverse outcomes. Methimazole, a clinically used antithyroid agent, is known to be an inhibitor of FMO3 [35], and may play a role in the prevention of FMO3 mediated cardiovascular damage. A larger study, with a sample size sufficient to conduct a full Mendelian randomization studies, is required in order to fully evaluate the association between FMO3 activity, TMAO, and mortality.

## Supporting Information

S1 Table Association of TMAO concentration, kidney disease progression and all-cause mortality. Plasma TMAO concentrations were not significantly associated with kidney disease progression or all-cause mortality, after adjustment for potential confounders.
(DOCX)

## Abbreviations

ACN: Acetonitrile
CKD: Chronic Kidney Disease
CRP: C-reactive protein
eGFR: Estimated glomerular filtration rate
FMO3: Flavin-containing monoxygenase 3
GEE: Generalized estimating equations
HDL: High-density lipoprotein
HILIC: hydrophobic interaction chromatography
LC-MS: Liquid chromatograpy—mass spectrometry
LDL: Low-density lipoprotein
SKS: Seattle Kidney Study
TMA: Trimethylamine
TMAO: Trimethylamine N-oxide

## References

[1] Shlipak MG, Sarnak MJ, Katz R, Fried LF, Seliger SL, Newman AB, et al Cystatin C and the risk of death and cardiovascular events among elderly persons. The New England journal of medicine. 2005;352(20):2049–60. Epub 2005/05/20. 10.1056/NEJMoa043161 . 15901858

[2] Sarnak MJ, Katz R, Stehman-Breen CO, Fried LF, Jenny NS, Psaty BM, et al Cystatin C concentration as a risk factor for heart failure in older adults. Annals of internal medicine. 2005;142(7):497–505. Epub 2005/04/06. . 15809461 10.7326/0003-4819-142-7-200504050-00008

[3] Anavekar NS, McMurray JJ, Velazquez EJ, Solomon SD, Kober L, Rouleau JL, et al Relation between renal dysfunction and cardiovascular outcomes after myocardial infarction. The New England journal of medicine. 2004;351(13):1285–95. Epub 2004/09/24. 10.1056/NEJMoa041365 . 15385655

[4] Tang WH, Wang Z, Kennedy DJ, Wu Y, Buffa JA, Agatisa-Boyle B, et al Gut microbiota-dependent trimethylamine N-oxide (TMAO) pathway contributes to both development of renal insufficiency and mortality risk in chronic kidney disease. Circulation research. 2015;116(3):448–55. Epub 2015/01/20. 10.1161/CIRCRESAHA.116.305360 25599331 PMC4312512

[5] Stubbs JR, House JA, Ocque AJ, Zhang S, Johnson C, Kimber C, et al Serum Trimethylamine-N-Oxide is Elevated in CKD and Correlates with Coronary Atherosclerosis Burden. Journal of the American Society of Nephrology: JASN. 2015 Epub 2015/08/01. ASN.2014111063 [pii] 10.1681/ASN.2014111063 . 26229137 PMC4696571

[6] Wang Z, Klipfell E, Bennett BJ, Koeth R, Levison BS, Dugar B, et al Gut flora metabolism of phosphatidylcholine promotes cardiovascular disease. Nature. 2011;472(7341):57–63. Epub 2011/04/09. 10.1038/nature09922 21475195 PMC3086762

[7] Craig SA. Betaine in human nutrition. Am J Clin Nutr. 2004;80(3):539–49. Epub 2004/08/24. 80/3/539 [pii]. . 15321791 10.1093/ajcn/80.3.539

[8] Lang DH, Yeung CK, Peter RM, Ibarra C, Gasser R, Itagaki K, et al Isoform specificity of trimethylamine N-oxygenation by human flavin-containing monooxygenase (FMO) and P450 enzymes: selective catalysis by FMO3. Biochem Pharmacol. 1998;56(8):1005–12. Epub 1998/10/17. S0006295298002184 [pii]. . 9776311 10.1016/s0006-2952(98)00218-4

[9] Bell JD, Lee JA, Lee HA, Sadler PJ, Wilkie DR, Woodham RH. Nuclear magnetic resonance studies of blood plasma and urine from subjects with chronic renal failure: identification of trimethylamine-N-oxide. Biochim Biophys Acta. 1991;1096(2):101–7. Epub 1991/02/22. . 2001424 10.1016/0925-4439(91)90046-c

[10] Treacy E, Johnson D, Pitt JJ, Danks DM. Trimethylaminuria, fish odour syndrome: a new method of detection and response to treatment with metronidazole. J Inherit Metab Dis. 1995;18(3):306–12. Epub 1995/01/01. . 7474897 10.1007/BF00710420

[11] Zhang AQ, Mitchell S, Smith R. Fish odour syndrome: verification of carrier detection test. J Inherit Metab Dis. 1995;18(6):669–74. Epub 1995/01/01. . 8750603 10.1007/BF02436755

[12] Mackay RJ, McEntyre CJ, Henderson C, Lever M, George PM. Trimethylaminuria: causes and diagnosis of a socially distressing condition. Clin Biochem Rev. 2011;32(1):33–43. Epub 2011/04/01. 21451776 PMC3052392

[13] Koukouritaki SB, Poch MT, Henderson MC, Siddens LK, Krueger SK, VanDyke JE, et al Identification and functional analysis of common human flavin-containing monooxygenase 3 genetic variants. J Pharmacol Exp Ther. 2007;320(1):266–73. Epub 2006/10/20. jpet.106.112268 [pii] 10.1124/jpet.106.112268 . 17050781

[14] Mao M, Matimba A, Scordo MG, Gunes A, Zengil H, Yasui-Furukori N, et al Flavin-containing monooxygenase 3 polymorphisms in 13 ethnic populations from Europe, East Asia and sub-Saharan Africa: frequency and linkage analysis. Pharmacogenomics. 2009;10(9):1447–55. Epub 2009/09/19. 10.2217/pgs.09.77. 10.2217/pgs.09.77 19761368

[15] Akerman BR, Lemass H, Chow LM, Lambert DM, Greenberg C, Bibeau C, et al Trimethylaminuria is caused by mutations of the FMO3 gene in a North American cohort. Mol Genet Metab. 1999;68(1):24–31. Epub 1999/09/10. 10.1006/mgme.1999.2885 S1096-7192(99)92885-8 [pii]. . 10479479

[16] Treacy E, Johnson D, Pitt JJ, Danks DM. Trimethylaminuria, fish odour syndrome: a new method of detection and response to treatment with metronidazole. Journal of inherited metabolic disease. 1995;18(3):306–12. Epub 1995/01/01. . 7474897 10.1007/BF00710420

[17] Zschocke J, Kohlmueller D, Quak E, Meissner T, Hoffmann GF, Mayatepek E. Mild trimethylaminuria caused by common variants in FMO3 gene. Lancet. 1999;354(9181):834–5. Epub 1999/09/15. . 10485731 10.1016/s0140-6736(99)80019-1

[18] Yeung CK, Adman ET, Rettie AE. Functional characterization of genetic variants of human FMO3 associated with trimethylaminuria. Arch Biochem Biophys. 2007;464(2):251–9. Epub 2007/05/29. 10.1016/j.abb.2007.04.014 17531949 PMC2039921

[19] Robinson-Cohen C, Littman AJ, Duncan GE, Roshanravan B, Ikizler TA, Himmelfarb J, et al Assessment of Physical Activity in Chronic Kidney Disease. J Ren Nutr. 2012 S1051-2276(12)00118-5 [pii] 10.1053/j.jrn.2012.04.008 . 22739659 PMC3496802

[20] Roshanravan B, Khatri M, Robinson-Cohen C, Levin G, Patel KV, de Boer IH, et al A prospective study of frailty in nephrology-referred patients with CKD. Am J Kidney Dis. 2012;60(6):912–21. 10.1053/j.ajkd.2012.05.017 22770927 PMC3491110

[21] Robinson-Cohen C, Littman AJ, Duncan GE, Weiss NS, Sachs MC, Ruzinski J, et al Physical Activity and Change in Estimated GFR among Persons with CKD. J Am Soc Nephrol. 2013 10.1681/ASN.2013040392 . 24335971 PMC3904564

[22] Echeverria D, Woods JS, Heyer NJ, Martin MD, Rohlman DS, Farin FM, et al The association between serotonin transporter gene promotor polymorphism (5-HTTLPR) and elemental mercury exposure on mood and behavior in humans. J Toxicol Environ Health A. 2010;73(15):1003–20. Epub 2010/06/09. 10.1080/15287390903566591 922753199 [pii]. 20526950 PMC2882654

[23] Woods JS, Heyer NJ, Russo JE, Martin MD, Farin FM. Genetic polymorphisms affecting susceptibility to mercury neurotoxicity in children: summary findings from the Casa Pia Children's Amalgam clinical trial. Neurotoxicology. 2014;44:288–302. Epub 2014/08/12. 10.1016/j.neuro.2014.07.010 S0161-813X(14)00139-9 [pii]. 25109824 PMC4176692

[24] Robinson-Cohen C, Littman AJ, Duncan GE, Weiss NS, Sachs MC, Ruzinski J, et al Physical activity and change in estimated GFR among persons with CKD. Journal of the American Society of Nephrology: JASN. 2014;25(2):399–406. Epub 2013/12/18. 10.1681/ASN.2013040392 ASN.2013040392 [pii]. 24335971 PMC3904564

[25] Levey AS, Stevens LA, Schmid CH, Zhang YL, Castro AF, 3rd, Feldman HI, et al A new equation to estimate glomerular filtration rate. Annals of internal medicine. 2009;150(9):604–12. Epub 2009/05/06. 19414839 10.7326/0003-4819-150-9-200905050-00006 PMC2763564

[26] Zeger SL, Liang KY. Longitudinal data analysis for discrete and continuous outcomes. Biometrics. 1986;42(1):121–30. Epub 1986/03/01. . 3719049

[27] Bandyopadhyay A, Saxena K, Kasturia N, Dalal V, Bhatt N, Rajkumar A, et al Chemical chaperones assist intracellular folding to buffer mutational variations. Nat Chem Biol. 2012;8(3):238–45. Epub 2012/01/17. 10.1038/nchembio.768 nchembio.768 [pii]. 22246401 PMC3527004

[28] Shih DM, Wang Z, Lee R, Meng Y, Che N, Charugundla S, et al Flavin containing monooxygenase 3 exerts broad effects on glucose and lipid metabolism and atherosclerosis. Journal of lipid research. 2015;56(1):22–37. Epub 2014/11/08. 10.1194/jlr.M051680 jlr.M051680 [pii]. 25378658 PMC4274068

[29] Warrier M, Shih DM, Burrows AC, Ferguson D, Gromovsky AD, Brown AL, et al The TMAO-Generating Enzyme Flavin Monooxygenase 3 Is a Central Regulator of Cholesterol Balance. Cell Rep. 2015 Epub 2015/01/21. S2211-1247(14)01065-1 [pii] 10.1016/j.celrep.2014.12.036 25600868 PMC4501903

[30] Bushueva O, Solodilova M, Churnosov M, Ivanov V, Polonikov A. The Flavin-Containing Monooxygenase 3 Gene and Essential Hypertension: The Joint Effect of Polymorphism E158K and Cigarette Smoking on Disease Susceptibility. Int J Hypertens. 2014;2014:712169 Epub 2014/09/23. 10.1155/2014/712169 25243081 PMC4163302

[31] Allerston CK, Shimizu M, Fujieda M, Shephard EA, Yamazaki H, Phillips IR. Molecular evolution and balancing selection in the flavin-containing monooxygenase 3 gene (FMO3). Pharmacogenet Genomics. 2007;17(10):827–39. Epub 2007/09/22. 10.1097/FPC.0b013e328256b198 01213011-200710000-00004 [pii]. . 17885620

[32] Dolan C, Shields DC, Stanton A, O'Brien E, Lambert DM, O'Brien JK, et al Polymorphisms of the Flavin containing monooxygenase 3 (FMO3) gene do not predispose to essential hypertension in Caucasians. BMC Med Genet. 2005;6:41 Epub 2005/12/06. 1471-2350-6-41 [pii] 10.1186/1471-2350-6-41 16324215 PMC1316875

[33] Treacy EP, Akerman BR, Chow LM, Youil R, Bibeau C, Lin J, et al Mutations of the flavin-containing monooxygenase gene (FMO3) cause trimethylaminuria, a defect in detoxication. Hum Mol Genet. 1998;7(5):839–45. Epub 1998/05/23. ddb113 [pii]. . 9536088 10.1093/hmg/7.5.839

[34] Kaysen GA, Johansen KL, Chertow GM, Dalrymple LS, Kornak J, Grimes B, et al Associations of Trimethylamine N-Oxide With Nutritional and Inflammatory Biomarkers and Cardiovascular Outcomes in Patients New to Dialysis. J Ren Nutr. 2015;25(4):351–6. Epub 2015/03/25. 10.1053/j.jrn.2015.02.006 S1051-2276(15)00065-5 [pii]. 25802017 PMC4469547

[35] Poulsen LL, Hyslop RM, Ziegler DM. S-oxidation of thioureylenes catalyzed by a microsomal flavoprotein mixed-function oxidase. Biochem Pharmacol. 1974;23(24):3431–40. Epub 1974/12/15. 0006-2952(74)90346-3 [pii]. . 4441423 10.1016/0006-2952(74)90346-3

