# Supplementary material for: Association of FMO3 Variants and Trimethylamine N-Oxide Concentration, Disease Progression, and Mortality in CKD Patients
Source: PLoS One. 2016 Aug 11;11(8):e0161074. doi: 10.1371/journal.pone.0161074 (PMC4981377; doi:10.1371/journal.pone.0161074)
Supplement: S1 Table — Plasma TMAO concentrations were not significantly associated with kidney disease progression or all-cause mortality, after adjustment for potential confounders. (DOCX) [file pone.0161074.s001.docx]

**S1 Table: Association of TMAO concentration, kidney disease progression and all-cause mortality**

| **TMAO Exposure (µg/mL)** | **Adjusted mean % change in eGFR per year (95% CI)** | | **Number of deaths** | **IR, per 1000 p-y** | **Adjusted* HR (95% CI)** | |
| --- | --- | --- | --- | --- | --- | --- |
|  | Model 1 | Model 2 |  |  | Model 1 | Model 2 |
| TMAO tertile |  |  |  |  |  |  |
| <0.94 | -3.52 (-7.83, +0.77) | -5.70 (-10.5, -0.94) | 8 | 25.7 | 1.0 (ref.) | 1.0 (ref.) |
| 0.94 – 1.71 | -37.6 (-46.5, -28.7) | -40.7 (-49.5, -31.8) | 13 | 36.7 | 1.19 (0.48, 2.97) | 0.57 (0.23, 1.44) |
| ≥ 1.71 | -56.4 (-63.1, -4.99) | -58.9 (-65.5, -52.2) | 24 | 70.9 | 2.45 (1.09, 5.50) | 1.25 (0.48, 3.28) |
| p-for-trend | 0.09 | 0.47 |  |  | 0.02 | 0.37 |

Model 1: Age, race, sex

Model 2: Model 1 + SBP, LDL, HDL, Smoking, crp (log), eGFR (log) (in mortality analyses only).
